# Expression, purification, and characterization of self-assembly virus-like particles of capsid protein L1 HPV 52 in *Pichia pastoris* GS115

**DOI:** 10.1186/s43141-023-00571-0

**Published:** 2023-11-20

**Authors:** Chindy Nur Rosmeita, Sri Budiarti, Apon Zaenal Mustopa, Ela Novianti, Sri Swasthikawati, Sheila Chairunnisa, Ai Hertati, Maritsa Nurfatwa, Nurlaili Ekawati, Nurhasni Hasan

**Affiliations:** 1 Research Center for Genetic Engineering, Research Organization for Life Sciences and Environment, National Research and Innovation Agency (BRIN), Bogor, 16911 Indonesia; 2 Program of Biotechnology, Graduate School, IPB University, Bogor, Indonesia; 3 Indonesia Research Center for Bioresources and Biotechnology, IPB University, Bogor, Indonesia; 4 Faculty of Pharmacy, Universitas Hasanuddin, Jl. Perintis Kemerdekaan Km 10, Makassar, 90245 Republic of Indonesia

**Keywords:** Capsid protein L1, HPV 52, Recombinant *Pichia pastoris*, Protein purification, VLP-based vaccine

## Abstract

**Background:**

Cervical cancer caused by the human papillomavirus (HPV) is one of the most frequent malignances globally. HPV 52 is a high-risk cancer-causing genotype that has been identified as the most prevalent type in Indonesia. Virus-like particles (VLP)-based vaccinations against HPV infection could benefit from self-assembled VLP of L1 capsid protein.

**Result:**

The recombinant HPV 52 L1 was expressed in *Pichia pastoris* on a shake-flask scale with 0.5% methanol induction in this study. The copy number was used to compare the expression level and stability. The colony that survived on a solid medium containing 2000 μg/ml of Zeocin was selected and cultured to express HPV 52 L1. DNA was extracted from the chosen colony, and the copy was determined using qPCR. HPV 52 L1 protein was then purified through fast performance liquid chromatography. Transmission electron microscopy (TEM) evaluation confirmed the VLP self-assembly. The genomic DNA remained intact after 100 generations of serial cultivation under no selective pressure medium conditions, and the protein produced was relatively stable. However, the band intensity was slightly lower than in the parental colony. In terms of copy number, a low copy transformant resulted in low expression but produced a highly stable recombinant clone. Eventually, the L1 protein expressed in *Pichia pastoris* can self-assemble into VLP. Therefore, recombinant HPV possesses a stable clone and the ability to self-assemble into VLP.

**Conclusion:**

The recombinant L1 HPV 52 protein is successfully expressed in *P. pastoris* within a size range of approximately 55 kDa and demonstrated favorable stability. The L1 protein expressed in *Pichia pastoris* successful self-assembled of HPV VLPs, thereby establishing their potential efficacy as a prophylactic vaccine.

## Background

Cervical cancer has emerged as a leading cause of cancer-related mortality in women, ranking fourth globally with a distribution of cases and death of 6.5% and 7.7%, with cancer cases often occurring in women aged between 15 and 44 years [1, 2]. In Indonesia, cervical cancer has surpassed breast cancer as the second-leading cause of death. There are 32,369 new cases of cervical cancer, with a mortality rate of 18,279 [3]. In developing countries, poverty and the number of children are the leading causes of the high incidence of cervical cancer [4]. Due to their elevated cancer risk, women of productive age should begin to prioritize their reproductive health.

Human papillomavirus (HPV) belongs to the Papovaviridae family; it is relatively small, 55 nm in diameter, and a non-enveloped virus and has an icosahedral capsid consisting of 72 capsomers [5]. HPV and its high-risk oncogenic types are the main pathogens that cause cervical cancer [6]. HPV infects epithelial cells involving the skin, respiratory mucosa, or genital tract. High-risk HPV is the type of HPV that causes malignant tumors to lead to cancer containing HPV 16, 18, 31, 33, 45, 51, and 52 [7]. Data from the Agency for Health Research and Development, Indonesian Ministry of Health, reported that HPV has the highest prevalence in Indonesia, namely HPV 16, 18, and 52, where HPV type 52 is a high-risk HPV with the highest percentage of 18.5% [8].

One method to prevent cancers caused by HPV is through the early vaccination of girls aged 9–14 as recommended by the WHO [9]. In recent years, many clinical trials have demonstrated that VLP-based HPV prophylactic vaccines can induce a long-lasting immune response against HPV infection [10]. A virus-like particle (VLP) has a similar morphology to that of HPV virions. However, because it lacks viral DNA and is not infectious, it provides a safe immune response because it does not stimulate harmful oncogenes. The assembly of capsid proteins from HPV L1 proteins generates VLPs [11, 12]. The HPV capsid protein is composed of the L1 protein, which accounts for around 80% of the total and is referred to as the major capsid protein, and the L2 protein, which is referred to as the minor capsid protein [13]. The ability of L1 to induce antibodies has been extensively studied, proving that L1 is a highly potential and effective vaccine candidate [14, 15].

HPV L1 protein has been expressed in several bacteria, such as *E. coli* [16], insect, mammalian, plant, and yeast cell expression systems [17–20]. The application of vaccines in developing countries with high HPV infection rates is limited by low production yields and relatively high costs. Therefore, low-cost vaccine production is required. Eukaryotic systems such as insect and mammalian cells have the disadvantage of low expression levels and slow growth rates. This system leads to high production costs and may hinder widespread HPV vaccination in developing countries.

Yeast protein expression system is widely employed in industrial-scale production, exemplified by the approved vaccines Gardasil® and Gardasil-9® [21]. In recent years, methylotrophic yeasts like *Pichia pastoris* have become a viable option for biopharmaceutical recombinant proteins [22]. This phenomenon occurs due to the yeast’ preference for utilizing methyl groups as a source of nutrition, as opposed to carbohydrates. The *P. pastoris* expression system was selected for heterologous protein expression due to its ability to utilize methanol as a carbon source and induce foreign protein expression through methanol use [23]. This system exhibits a high expression rate, rapid growth rate, and cost-effective and employs advanced fermentation technology. *P. pastoris* possesses promoter derived from the AOX1 gene, which is well-suited for regulating the expression of foreign genes. Furthermore, this yeast exhibits physiological properties that enable it to produce a high cell density compared to other fermentative yeasts [24].

Previously, the L1 HPV 52 protein was produced in the *P. pastoris* expression system, but only in strain BG10 [25], and it was not purified. This strain is now part of the National Research and Innovation Agency’s (BRIN) collection. Therefore, the present study was conducted on a different strain, namely *P. pastoris* strain GS115 (Mut^+^), which has a faster growth rate and a high metabolic rate, resulting in higher productivity. In Indonesia, cervical cancer cases caused by HPV 52 are the focus of national vaccination program. Consequently, the development of HPV vaccine targeting type 52 is a logical step in combating against this global pathogen. In addition, *P. pastoris* is a cost-effective and volume-efficient alternative to superior eukaryotic expression systems for VLP production. Currently, available vaccines are still not accessible to the majority of the economically disadvantaged population in Indonesia, as the commercial HPV vaccine costs at least US $492.32 for one person (three doses). In order to reduce the incidence of cervical cancer in developing countries, particularly Indonesia, there is a need for a strategy to generate a less expensive HPV vaccine that the government can provide to everyone.

Herein, we describe the design and construct of a recombinant L1 HPV 52 protein, as well as its production and purification by fast performance liquid chromatography (FPLC), and the subsequent characterization of the L1 HPV 52 structural protein in *P. pastoris*. This research can provide alternatives and broader perspectives on tackling the HPV 52 pathogen by producing VLP-based vaccines urgently needed in developing countries.

## Methods

### **Yeast and bacterial strains, plasmid, and media**

The methylotrophic yeast *P. pastoris* GS115 strain and pD902-HPV 52 as a vector expression (ATUM, CA, USA) were used in this study. The *Escherichia coli* BL21 DE3 strain (Invitrogen, Waltham, USA) was used as the host for plasmid propagation.

Low-salt LB medium is as follows: 1% tryptone, 0.5% NaCl, and 0.5% yeast extract at pH 7.5; YPD: 1% yeast extract, 2% peptone, and 2% dextrose; YPDS: 1% yeast extract, 2% peptone, 2% dextrose, and 1-M sorbitol; BMGY: 1% yeast extract, 2% peptone, 100-mM potassium phosphate buffer pH 6, 1.34% yeast nitrogen base (YNB) without amino acid, 1% glycerol, and 0.2% biotin; and BMMY: 1% yeast extract, 2% peptone, 1.34% YNB, 100-mM potassium phosphate buffer pH 6.0, 0.2% biotin, 0.5% methanol as *P. pastoris* pre-induction growth medium, and induction medium, respectively.

### Design and construction of recombinant plasmids

The HPV 52 L1 gene was designed according to our previous study [25]. The gene was codon-optimized for *Pichia pastoris* and commercially synthesized by ATUM Company DNA 2.0 Gene Design & Synthesis (California, USA). It was then inserted into pD902 between *EcoRI* and *NotI* sites and was copied by *Escherichia coli*, which persisted at 25 μg/ml Zeocin on a low-salt LB medium. The gene was amplified by a polymerase chain reaction (PCR, Axygen MaxyGene II, USA) using the specific primers shown in Table 1. The PCR conditions used were pre-denaturation (95 °C for 5 min), 40 cycles of denaturation (95 °C for 1 min), annealing (55 °C for 30 s), extension (72 °C for 1 min), and post-extension (72 °C for 5 min).

**Table 1:** List of primers used

Primer’s name	Usability	Sequence 5′-3′	Amplicon size (bp)
RT-PCR L1 HPV-52 F	Stability test	TTG-TCG-ATT-CCA-GGC-TTC-CC	96
RT-PCR L1 HPV-52 R		GCC-CCT-CGG-TGT-TTG-TAT-TT	
HPV-52 yeast F	Gene amplification	GAA-TTC-AAA-ACG-ATG-TCA-GTT-TGG-C	1514
HPV-52 yeast R		CAA-ATG-TTC-GCA-TTC-TGA-CAT-CCT-CTT-GAG-C	
AOX1_forward	Analysis	GAC-TGG-TTC-CAA-TTG-ACA-AGC	-
AOX1_reverse	Integration	GCA-AAT-GGC-ATT-CTG-ACA-TCC	-

### Transformation of yeast and selection of multi-copy

Recombinant plasmids were extracted and linearized using the *SacI* enzyme (Thermo Scientific, MA, USA) to transform *P. pastoris*. The linear plasmid pD902-L1HPV 52 was purified with the QIAquick Gel Extraction Kit (Qiagen, Germany). The transformation was performed using the Groningen method [26], with slight modifications. Competent cell mixtures were pulsed using standard settings for *P. pastoris* from the Gene Pulser Xcell electroporation device (Bio-Rad, USA) at 7.5 kV/cm, 50 μF, and 129 Ω. Transformants were cultured on YPDS agar plates containing 100 μg/mL Zeocin and incubated for 3–5 days at 28 °C. Transformant colonies were screened on YPD agar plates containing a Zeocin concentration gradient (100–1000 μg/mL). Genes from colonies that survived the highest Zeocin concentration were extracted, and PCR amplification was performed, followed by gene sequencing analysis. Amplification conditions were performed the same as described above.

### Expression of recombinant protein

Recombinant *P. pastoris* cells were grown in test tubes containing 5-mL YPD and 100 μg/mL Zeocin and incubated at 30 °C with shaking at 250 rpm until OD600 reached ~2–6. Then, 1 mL of culture was inoculated into 25 mL of BMGY medium and incubated under the same conditions for 24 h with 1:10 aeration to the total volume. After incubation, cells were centrifuged at 3000 × g at 4 °C for 5 min, resuspended in BMMY medium, and incubated at 30 °C with shaking at 250 rpm. To induce expression of recombinant proteins from *P. pastoris*, 0.5% (v/v) methanol was added every 24 h, followed by a culture sampling to analyze optical density.

Cells were harvested after 72 h of growth, centrifuged at 3000 × g for 5 min, and lysed according to previously reported methods [27], with slight modifications. Harvested cultures were resuspended in disruption buffer (50-mM sodium phosphate, 1-mM EDTA, 5% glycerol, 1-mM phenylmethylsulfonyl fluoride (PMSF), pH 7.4), mixed with an equal volume of 0.5-mm diameter-glass beads (Sigma-Aldrich, UK), and lysed by vortexing for 30 s, followed by incubating on ice for 30 s (eight cycles). The clear supernatant was collected by centrifuging the mixture at 12,000 rpm for 15 min at 4 °C and stored at −20 °C for further analysis.

### Sodium dodecyl sulfate polyacrylamide gel electrophoresis (SDS-PAGE) and Western blotting analysis

The characterization of HPV 52 L1 expression levels in *P. pastoris* was analyzed by SDS-PAGE and Western blotting. Samples were mixed with Laemmli sample buffer and denatured for 10 min at 95 °C. Proteins were fractionated on 10% SDS-PAGE and stained with Coomassie brilliant blue [28]. Afterwards, Western blotting with the indicated antibodies followed the standard protocol. Fractionated proteins were transferred to nitrocellulose membranes with Bio-Rad Mini Trans-BlotVR (Bio-Rad, USA) at 400 mA per gel for 100 min in transfer buffer (25-mM Tris-base, 192-mM glycine, 20% methanol). Membranes were blocked with Tris-buffered saline and 0.1% Tween 20 (TBST) supplemented with 5% skim milk for 1 h, washed with TBST, and incubated with specific antibodies for 2 h at room temperature. Rabbit anti-HPV 52 L1 antibody (1:2000 dilution, Creative Diagnostics, USA) and goat alkaline phosphatase-conjugated anti-rabbit IgG antibody (1:2000 dilution, Invitrogen, USA) were used as primary and secondary antibodies, respectively. After incubation, the membrane was washed with TBST, and proteins were visualized using AP NBT/BCIP chromogenic substrate (Thermo Fisher Scientific, USA).

### Stability analysis of recombinant HPV-52 L1 clones

Recombinant *P. pastoris* HPV 52 clones were inoculated into 2 mL of YPD containing 100 μg/mL Zeocin and grown at 30 °C and 250 rpm for 18–24 h to get ~2 × 10^7^ cells/mL. Two subcultures of 10 μL were made, with one of the media containing a selection marker while the other did not. From the grown culture, serial dilutions were made and spread on nonselective and selective solid medium, incubated at 30 °C for 2–3 days [29]. New subcultures were made from the latest generation and repeated for 100 generations. The prototrophic colonies were observed and calculated on the selective versus the nonselective plate, and all colonies were extracted to obtain gDNA and confirm the L1 sequence of all generations. From the parental cultures, five subsequent subcultures were made from the 20, 40, 60, 80, and 100 generations, consecutively, each in 10 mL of BMMY medium. The cultures were induced with 0.5% methanol, and the expression of HPV L1 was analyzed on a Western blot.

### Bicinchoninic acid (BCA) assay and enzyme-linked immunosorbent assay (ELISA)

The total protein concentration was quantified using the Pierce BCA assay (Thermo Fisher Scientific, USA), following the manufacturer’s protocols with slight modifications. An anti-L1 HPV 52 antibody (Creative Diagnostics, USA) was used as the positive control. The results were calculated based on the standard curve, with three replicates of each sample analyzed.

The HPV 52 L1 protein was examined by ELISA using specific antibodies (Creative Diagnostics, USA). Briefly, the ELISA plate was coated with the anti-L1 HPV 52 antibody (1:5000) overnight at 4 °C. The coated plate was washed three times with washing buffer (PBST; 0.05% Tween-20 in PBS) and blocked with blocking buffer (7.5% bovine serum albumin in PBST) for 1 h at room temperature. Unabsorbed proteins were removed by washing buffer. Samples were then added to the plate for 2-h incubation at room temperature. After being washed three times with wash buffer, the anti-HPV 52 L1 (1:1000) as a primary antibody was added and incubated for another 2 h at room temperature. The unbound primary antibodies were removed by washing. The anti-mouse IgG-HRP conjugate (1:2000 dilution, Life Technologies, USA) was added to the well, incubated for 2 h at room temperature, and washed with wash buffer. The proteins were stained with the substrate ABTS (Thermo Fisher Scientific, USA), and the optical density was detected using a microplate reader (Varioskan, Thermo Fisher Scientific, USA) at 602 nm.

### Transmission electron microscopy (TEM)

HPV 52 L1 VLP formation was analyzed using TEM. The precipitated L1 protein was dialyzed with PBS prior to TEM preparation. Samples were then absorbed on carbon-coated grids and negatively stained with 2% phosphotungstic acid [30]. Samples were visualized using an electron microscope (JEM JEOL 1010, JEOL, Japan) at 30,000× magnification with an accelerating voltage of 80 kV at the Eijkman Institute of Molecular Biology (Jakarta, Indonesia).

### Strong cation exchange chromatography

Cation exchange chromatography, as part of fast performance liquid chromatography (FPLC), was used as the first step of purification. The sample was filtered with 0.45 μm and then purified with HiTrap Capto SP ImpRes column 5 × 5 mL (product no. 17546855, Cytiva, Sweden) on ÄKTA Pure (GE HealthCare, USA). After the sample application was complete, the column was washed with five column volumes of binding buffer. The column was eluted with a linear gradient from 100% binding buffer (50-mM MOPS Na^+^, 20-mM NaCl, pH 6.2) to 100% elution buffer (50-mM MOPS Na^+^, 1-M NaCl, pH 6.2). The total volume of the gradient was 20 column volumes. The fractions were collected and analyzed for HPV 52 L1 protein using SDS-PAGE and Western blotting.

### Size-exclusion chromatography

The fractions containing HPV 52 L1 protein from the first step of purification were collected, and 0.03% Tween-80 was added and then purified with HiPrep 16/60 Sephacryl™ S-100 HR 120 mL (product no. 17-1165-01, GE HealthCare, Sweden) on ÄKTA pure (GE HealthCare, USA) at 5 mL/min, which was equilibrated with ten column volumes with running buffer. The running buffer for this column was 50-mM MOPS Na^+^ at pH 6.2, 500-mM NaCl, and 0.03% Tween-80. The fractions were collected and analyzed for HPV 52 L1 protein using SDS-PAGE and Western blotting.

## Result

### **Transformation and selection of multi-copy colonies**

On YPDS solid media containing Zeocin, transformed colonies were observed after 5 days of incubation. The absence of colonies on the solid medium containing Zeocin as compared to the solid medium lacking Zeocin as the positive control indicates that cell viability was in good condition. Colonies on solid media containing Zeocin indicated that pD902 carrying the L1 gene was effectively introduced into *P. pastoris*, as indicated by the presence of these colonies. Using the universal primer AOX (Invitrogen) and a specific primer for the HPV 52 L1 gene, the transformant colonies were confirmed via PCR and sequencing. The L1 gene was detected at 1500 bp with both the AOX primer and the HPV 52 L1-specific primer in PCR products performed on an agarose gel. The result of sequencing also revealed sequences corresponding to the target gene. Following a multi-copy colony selection with graded Zeocin concentrations, three colonies, clones 6, 7, and 15, survived and grew well up to 2000 μg/mL Zeocin. Three clones were then utilized to optimize the expression of L1 recombinant proteins.

### Expression of L1 HPV 52 in P. pastoris methanol induced

**Fig. 1:**
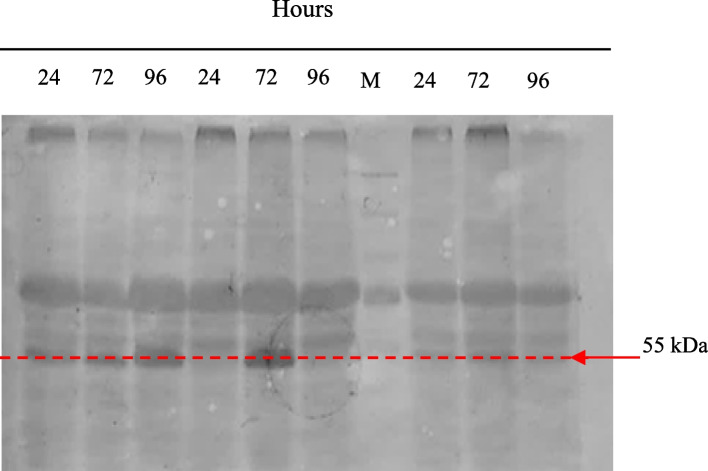
Western blot analysis of HPV 52 L1 expression in *Pichia pastoris* induced with 0.5% methanol at 48, 72, and 96 h of survived colonies on 2000 μg/ml Zeocin

Simultaneously, a *P. pastoris* host lacking an insert (serving as a negative control) and a *P. pastoris* carrying recombinant HPV 52 L1 were expressed and stimulated with 0.5% methanol for 96 h at 30 °C. Western blotting was used to characterize the collected samples. L1 protein was observed to have a size of approximately 55 kDa in the recombinant clone. On Western blotting, the 55-kDa protein reacted with an anti-rabbit HPV 52 L1 polyclonal antibody (Fig. 1).

### Stability analysis of recombinant P. pastoris HPV 52

**Fig. 2:**
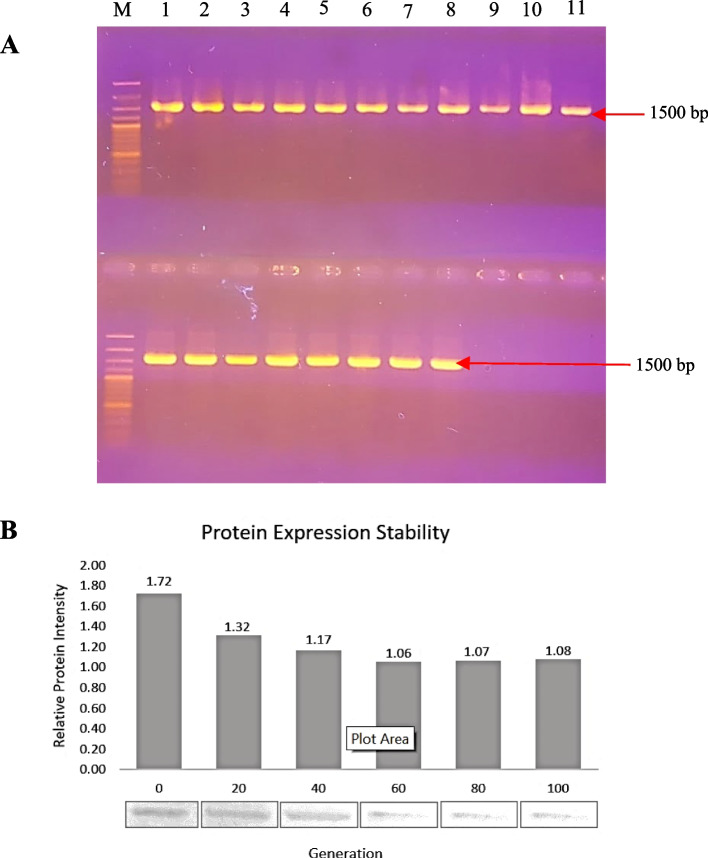
Stability analysis of the recombinant clone *P. pastoris*. **A** Genome DNA of 100 generations from selected clones showed persistent bands at 1500 bp, indicating a genetically stable clone. **B** Relative L1 protein intensity expressed from 100 generations indicated relative protein expression stability

The stability of recombinant HPV 52 L1 clones in *P. pastoris* was evaluated based on the analysis of inheritance, gDNA changed over 100 generations, and L1 expression in six groups in sequence. The stability of recombinant HPV 52 L1 clones in *P. pastoris* was determined by observing how they were transmitted, how the gDNA altered over 100 generations, and how the L1 gene was expressed in six distinct groups in sequence. The recombinant HPV 52 clone exhibited stable inheritance in nonselective marker media for the first 60 generations of 100 generations, after which the inheritance efficiency decreases marginally. On an agarose gel, PCR results of gDNA from 100 generations revealed a precise band at 1500 bp, indicating that pD902 carrying HPV 52 L1 had been irrevocably integrated into the genome of *P. pastoris* (Fig. 2). Using Western blots, the stability of L1 protein expression was evaluated. In the first three groups of six consecutive protein expressions, a distinct 55-kDa protein band was observed, whereas the remaining three groups exhibited uncertain narrow protein bands.

### Purification of HPV 52 VLP

**Fig. 3:**
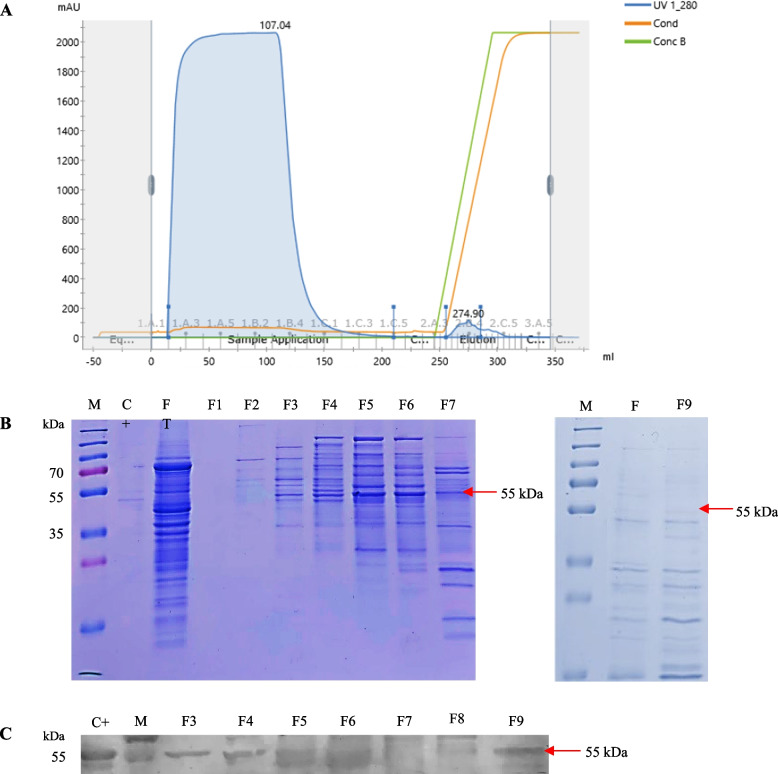
Purification of HPV 52 L1 by cation exchange. **A** Cation exchange chromatogram, **B** SDS-PAGE analysis, and **C** Western blot analysis of the HPV 52 L1 protein in the fractions collected from the cation exchange column. Lane M, molecular weight markers; lane C+, control of HPV 52 L1 designed by Creative Diagnostics (USA); lane FT, flowthrough; and lanes F1–F9, fractions from the elution peak. The arrows on the right in **B** and **C** indicate the position of HPV 52 L1

**Fig. 4:**
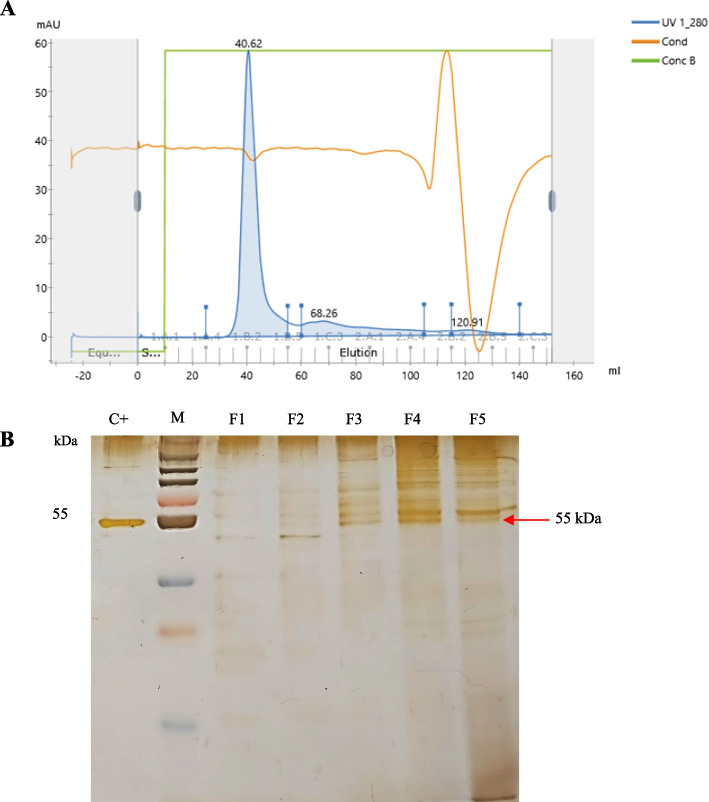
Purification of HPV 52 L1 by size exclusion. **A** Size-exclusion chromatogram. **B** SDS-PAGE analysis of the HPV 52 L1 protein in the fractions collected from the size-exclusion column. Lane M, molecular weight markers; lane C+, control of HPV 52 L1 that was designed by Creative Diagnostics (USA); lanes F1–F5, fractions from the elution peak. The arrows on the right in **B** indicate the position of HPV 52 L1

The yeast lysate was clarified and loaded into a cation exchange chromatography column. The chromatography curve for the separation of L1 HPV and 52 VLP from monomers and pentamers in elution fractions is depicted in Fig. 3a. SDS-PAGE analysis revealed that fractions 3–7 had higher purity than fractions 1–2 and 8–9 (Fig. 3B). Using Western blotting, the 55-kDa *HPV 52* L1 protein was detected in fractions 3–7 (Fig. 3C). Fractions 3–7 were collected and concentrated prior to the next purification. In size-exclusion chromatography, fractions from the first chromatography were pooled. Three peaks emerged on the chromatogram (Fig. 4A), and the highest peak was analyzed by SDS-PAGE with silver stain. The L1 HPV 52 protein was detected in fractions 4 and 5 measured 55 kDa in size (Fig. 4B). The amount of L1 HPV 52 protein at each purification stage was quantified using BCA Protein Assay Kit to determine total protein and an anti-HPV 52 L1 monoclonal antibody (Creative Diagnostics, USA) ELISA to determine total L1 protein. The recovery yield was determined to be 100% based on the total amount of HPV 52 L1 protein in the lysate supernatant.

### TEM analysis

The HPV 52 L1 protein expressed in *P. pastoris* was purified by ammonium sulfate precipitation, and 50-nm VLPs were observed by transmission electron microscopy. This study demonstrates that recombinant HPV 52 L1 can self-assemble into VLPs of the same size as natural virions.

## Discussion

HPV infection is a pathological condition that is closely linked to development of cervical cancer, which can be fatal. Vaccination against HPV infection is the most effective cervical cancer prevention [27, 29]. As these viruses cannot be cultured using cell culture techniques, prophylactic vaccines are being developed against HPV. Expression vectors such as eukaryotic cells that express the L1 protein encoding the viral capsid and self-assemble into VLPs are utilized in the production of HPV vaccines using recombinant DNA technology. VLPs display morphological and immunological characteristics similar to those of the natural HPV virus. However, they are incapable of causing infection and illness because they lack infectious DNA [17, 28].

In this study, we investigated the expression of HPV 52 L1 in *P. pastoris* using an integrative vector so that L1 protein could be produced. Colony transformants with high and stable expression levels are ideal and beneficial for large-scale protein production. Multi-copy transformants produce higher yields than transformants with a small number of copies [31]. In addition, the vector copy number is one of the most influential factors affecting intracellular expression yield [30, 31]. Increasing concentration of Zeocin selection was utilized in this study to obtain multi-copy transformants. This study generated three colonies that thrived on solid media containing 2000 μg/ml (results not shown). In fact, of the three selected colonies, L1 only expressed on BMGY and BMMY media induced with 0.5% methanol and produced a relatively thick band detected by Western blotting (Fig. 1). Analysis of copy number revealed that the selected colonies had a low copy number, suggesting that low copy may be one of the factors contributing to low expression yield. This result aligns with findings from other studies [32, 33] that copy number affects expression levels.

*Pichia pastoris* is known to have high stability due to chromosome integration that avoids segregation instability [34]. Based on the same profile of PCR visualization of genomic DNA extracted from selected recombinant clones, this study’s stability analysis showed that 100 generations of transformant colonies grown in a row under nonselective conditions were genetically stable. Furthermore, the protein expression stability of transformant clones was examined under shake flask conditions. At 20 to 100 generations of cultivation without selective pressure, selected recombinant clones exhibited stable expression based on relative protein band intensity induced by 0.5% methanol. Based on the protein concentration, there appeared to be a relative decrease compared to that of the progenitor colonies (Fig. 2). The reason for this decrease is unknown. Nonetheless, it is believed to be due to the lack of selective pressure maintenance. In his investigation, Aw (2012) [35] discovered that maintaining Zeocin as a selective pressure could improve the stability of multi-copy clones. However, the absence of Zeocin did not contribute to an overall loss of the expression vector [36].

The previous study examined the correlation between copy number and stability, revealing low copy transformants exhibit high stability when methanol induction is disregarded. In contrast, the stability of high copies is contingent upon the presence of methanol, as previous research has indicated that a significant decrease in stability occurs upon methanol induction as a result of metabolic burden [37]. This study elucidates the rationale for the observed low expression levels in the low copy transformants (two to three copies), which exhibited a degree of stability. The overexpression of heterologous proteins might induce strain in the secretion pathway, hence intensifying the existing protein responses that result in protein degradation and other cellular stress responses [38].

Purification facilitates the complex assembly reaction of the L1 protein into VLPs. VLPs of purified HPV L1 protein have excellent antigenicity and immunogenicity. The purification process has an effect on how antibodies are made to recognize the L1 capsid protein of HPV 52 [39]. The parameter that needs to be considered to determine whether the target protein is a good vaccine candidate is the molecular weight calculation. This study showed that our vaccine candidate possesses 55-kDa molecular weight. Proteins with a molecular weight of less than 100 kDa are considered as excellent candidates for vaccines [40]. Knowing the target protein’s isoelectric point (pI) value can help optimize the protein purification method, namely cation exchange chromatography. The pI value affects the binding of a protein ion with its charge medium, buffer solution, and column type and plays an important role in protein stability [41]. According to reports, pI and pH values correlate and can be used to determine the required pH during purification process [42]. The pI of the L1 protein of HPV 52 is 8.2, and the pH of the buffer used in the purification protocols was 6.2. The L1 HPV protein was successfully separated and purified using cation exchange chromatography, which is considered the most suitable method for protein or peptide separation [43].

Size-exclusion chromatography, which is commonly used in protein aggregation analysis for peptide and protein biotherapeutics [44], is the next purification step after ion exchange. In the process of protein purification, size-exclusion chromatography removes residual contaminants [45]. A pI values far from pH can increase protein aggregation due to the protein’s open structure [46]. By increasing the NaCl concentration and using MOPS buffer, the level of protein aggregation can be reduced. Low temperatures during the purification process inhibit the formation of [47]. HPV VLPs purified from yeast cells are typically. The nonionic surfactant polysorbate 80/Tween-80 in the buffer solution can therefore help stabilize HPV VLPs during reconstitution, freezing, and mechanical stress [48].

**Fig. 5:**
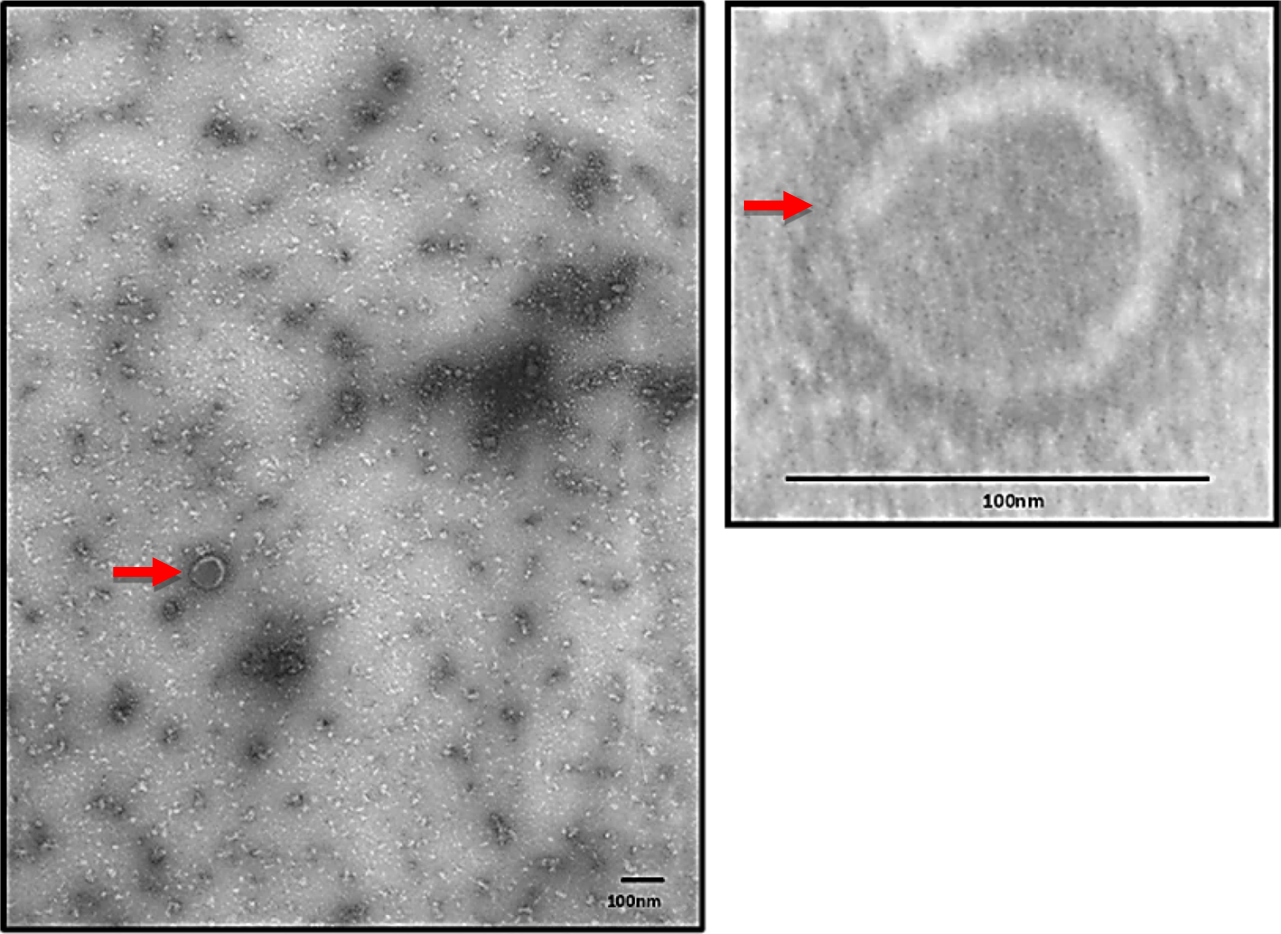
TEM analysis of L1 proteins. VLP is pointed by red arrow with a reference bar in the right-down corner of the box

The self-assembly of VLP with a diameter of 50–100 nm is observed when expressed L1 HPV 52 protein expressed, precipitated with ammonium sulfate, is used. This self-assembly occurs without any additional processing steps, as depicted in Fig. 5. The formation of VLPs within *P. pastoris* cells or their occurrence during ammonium sulfate precipitation remains uncertain. Another study revealed that VLPs originating from *P. pastoris*-derived HBsAg were assembled during the purification step [49]. The production of *P. pastoris*-derived VLPs may be a potential problem in strategic approaches. However, the findings of this work demonstrate that the expression of L1 HPV 52 in *P. pastoris* can result in the production of self-assembled VLPs, which have the potential to be utilized as a prophylactic vaccine.

## Conclusion

In conclusion, the recombinant L1 HPV 52 protein is successfully expressed in *P. pastoris* using 0.5% methanol induction and demonstrated favorable stability, making it a viable candidate for the generation of HPV vaccine. The L1 HPV 52 protein was obtained within a size range of approximately 55 kDa, rendering it a promising target for vaccine development. This study demonstrated the successful self-assembled of HPV VLPs, thereby establishing their potential efficacy as a prophylactic vaccine.

## Data Availability

All data generated or analyzed during this activity are included in this published article.

## Abbreviations

HPV: Human papillomavirus
VLP: Virus-like particles
TEM: Transmission electron microscopy
DNA: Deoxyribonucleic acid
BRIN: Badan Riset dan Inovasi Nasional/National Research and Innovation Agency
FPLC: Fast performance liquid chromatography
YPD: Yeast extract, peptone, and dextrose
YPDS: Yeast extract, peptone dextrose, and sorbitol
BMGY: Buffered complex glycerol *medium*
YNB: Yeast nitrogen base
BMMY: Buffered Methanol-complex *Medium*
SDS-PAGE: Sodium dodecyl-sulfate polyacrylamide gel electrophoresis
TBST: Tris-buffered saline and 0.1% Tween 20
BCA: Bicinchoninic acid
ELISA: Assay and enzyme-linked immunosorbent assay
PBST: Phosphate buffered saline and Tween
PCR: Polymerase chain reaction
EDTA: Ethylenediaminetetraacetic acid
PMSF: Phenylmethylsulfonyl fluoride
AP: Alkaline phosphatase
NBT/BCIP: Nitro blue tetrazolium/5-bromo-4-chloro-3-indolyl phosphate
IgG-HRP: Immunoglobulin G-horseradish peroxidase
ABTS: 2,2′-Azinobis [3-ethylbenzothiazoline-6-sulfonic acid]
